# Sodium Butyrate Therapy in Ulcerative Colitis: A Systematic Review of Preclinical and Clinical Evidence

**DOI:** 10.3390/medsci14040469

**Published:** 2026-08-09

**Authors:** George Ionuț Golea, Radu Alexandru Ilieș, Alexandra Caziuc, David Andraș, Alexandru Ilie-Ene, Radu Seicean, Radu Mircea Neagoe, Ștefana Dăscălescu, Luca Simionescu, Ștefan Cristian Vesa, George Călin Dindelegan, Florin Zaharie

**Affiliations:** 1 “Iuliu Hațieganu” University of Medicine and Pharmacy, 400012 Cluj-Napoca, Romania; george.ionu.golea@elearn.umfcluj.ro (G.I.G.); ilie_ene_alexandru@elearn.umfcluj.ro (A.I.-E.); luca.simionescu@elearn.umfcluj.ro (L.S.); 2 Department of General Surgery, First Surgical Clinic, Emergency County Hospital Cluj, 400006 Cluj-Napoca, Romania; andrasdavid88@elearn.umfcluj.ro (D.A.); rseicean@yahoo.com (R.S.); george.dindelegan@umfcluj.ro (G.C.D.); 3 Department of Surgery, Surgery Clinic I, “Iuliu Hațieganu” University of Medicine and Pharmacy, 400012 Cluj-Napoca, Romania; 4 Department of Surgery, “George Emil Palade” University of Medicine, Pharmacy, Science and Technology, 540139 Târgu Mureș, Romania; neagoerm@gmail.com; 5 Department of Medical Oncology, Institute of Oncology “Prof. Dr. Ion Chiricuță”, 400015 Cluj-Napoca, Romania; dascalescustefana28@gmail.com; 6 Department of Pharmacology, Toxicology and Clinical Pharmacology, “Iuliu Hațieganu” University of Medicine and Pharmacy, 400012 Cluj-Napoca, Romania; stefanvesa@gmail.com; 7 Department of General Surgery, Regional Institute of Gastroenterology and Hepatology “Octavian Fodor”, 400636 Cluj-Napoca, Romania; florinzaharie@yahoo.com; 8 Department of Surgery, Surgery Clinic III, “Iuliu Hațieganu” University of Medicine and Pharmacy, 400012 Cluj-Napoca, Romania

**Keywords:** ulcerative colitis, inflammatory bowel disease, sodium butyrate, short-chain fatty acids, gut microbiota, remission, systematic review

## Abstract

**Background/Objectives**: Sodium butyrate, a short-chain fatty acid produced by gut microbial fermentation of dietary fiber, has attracted increasing interest as a potential adjunctive therapy for ulcerative colitis (UC) because of its anti-inflammatory, barrier-protective, and immunomodulatory properties. This systematic review aimed to evaluate the current clinical and preclinical evidence regarding the efficacy and mechanisms of sodium butyrate in UC. **Methods**: A systematic review was conducted in accordance with the PRISMA 2020 guidelines and registered in PROSPERO (CRD420261437741). PubMed/MEDLINE, Scopus, Web of Science, ClinicalTrials.gov and the WHO International Clinical Trials Registry Platform were searched from February through April 2026. Eligible clinical and experimental studies investigating oral or rectal administration of sodium butyrate in UC or experimental colitis were included. Risk of bias was assessed using RoB 2, ROBINS-I, or the SYRCLE tool according to study design. **Results**: Twenty-two studies were included, comprising ten clinical trials, one prospective observational study and eleven preclinical animal studies. In adults diagnosed with UC, oral sodium butyrate, particularly microencapsulated formulations, demonstrated encouraging effects as an adjunct to standard therapy by improving clinical remission rates, quality of life and reducing disease activity and inflammatory biomarkers. Evidence in pediatric inflammatory bowel disease remained inconsistent. Studies evaluating rectal sodium butyrate enemas reported variable clinical efficacy despite evidence of local biological activity. Preclinical studies consistently demonstrated reduced intestinal inflammation, improved epithelial barrier integrity, modulation of oxidative stress, favorable alterations of the gut microbiota, and regulation of several mechanistic pathways identified across individual preclinical studies, including PI3K/AKT/mTOR, WNT/ERK, ERK/STAT3, autophagy and ferroptosis. **Conclusions**: Current evidence suggests that sodium butyrate represents a promising adjunctive therapeutic strategy for UC, supported by consistent mechanistic findings and encouraging clinical results, particularly with oral formulations. However, the available clinical evidence remains heterogeneous, and larger, well-designed randomized controlled trials with standardized formulations and treatment protocols are required before its routine clinical implementation can be considered.

## 1. Introduction

Ulcerative colitis (UC) represents a chronic inflammatory disease whose incidence is continuously increasing worldwide and growing evidence links it to gut microbiota and their metabolites, especially butyrate, which is a short-chain fatty acid (SCFA) synthesized by bacterial fermentation of fiber [1,2,3,4]. Recent research has investigated SCFAs not only as mechanistic contributors to mucosal health but also as potential adjunctive therapeutic approaches for UC and, more broadly, for inflammatory bowel disease (IBD) [4,5,6].

The pathogenesis of UC is multifactorial, involving genetic susceptibility, epithelial barrier dysfunction, dysregulated immune responses, environmental factors, and alterations of the gut microbiome [1]. Mucosal inflammation starts in the rectum and can extend proximally in a continuous pattern [2]. Barrier dysfunction and altered interactions between the mucus layer and the microbiota are now regarded as central mechanisms; hence, some authors describe UC as a “barrier organ disease” [1,7,8].

SCFAs (including acetate, propionate and butyrate) represent major microbial fermentation products of dietary fibers and are considered key energy sources for intestinal epithelial cells, while also playing major roles in the regulation of barrier integrity, motility, host metabolism, and immune responses [4,5,9].

Among them, butyrate has a particularly prominent role, ensuring colonocyte energy metabolism and maintaining integrity of junctions; moreover, it exerts immunomodulatory effects via mechanisms involving G protein-coupled receptors (like GPR41, GPR43, and GPR109A) as well as histone deacetylase inhibition [5,9,10]. In UC and other inflammatory bowel diseases, decreased abundance of butyrate-producing bacteria and reduced fecal SCFA levels are frequently observed, contributing to dysbiosis and mucosal inflammation [4,10,11].

In addition, butyrate promotes goblet cell differentiation and mucin production, supporting mucus barrier repair through mechanisms involving macrophage polarization toward an M2 phenotype and activation of WNT/ERK signaling pathways, which enhance MUC2 expression and other goblet cell markers [4,10]. It is also responsible for anti-inflammatory effects by reducing pro-inflammatory cytokine production and modulating both innate and adaptive immune responses at not only local but also systemic levels [5,10,11].

In this review, we aim to evaluate both the available clinical and mechanistic evidence regarding sodium butyrate in ulcerative colitis, with particular emphasis on its therapeutic efficacy, biological mechanisms of action and its potential role as an adjunctive treatment, as reported in the literature. Furthermore, we seek to assess their potential role as adjunctive therapeutic interventions in the current medical practice, highlighting efficacy and feasibility of use in management, rather than detailed mechanistic findings.

## 2. Materials and Methods

### 2.1. Search Strategy

This study was designed as a systematic review conducted in accordance with the PRISMA 2020 guidelines (Supplementary S1) [12]. This systematic review was registered in PROSPERO (International Prospective Register of Systematic Reviews) with registration number CRD420261437741.

A systematic literature search was performed in three bibliographic databases, PubMed, Web of Science, and Scopus, and the trial registries ClinicalTrials.gov and the WHO International Clinical Trials Registry Platform (ICTRP) were searched in order to identify studies evaluating the role of sodium butyrate in ulcerative colitis, with the last search run in April 2026 for each database.

The search combined two concept blocks, population (ulcerative colitis/inflammatory bowel disease/experimental colitis) and intervention (butyrate/butyric acid), using controlled vocabulary (MeSH) together with free-text title/abstract terms and truncation. The search strings were structured as presented in Table 1.

Only studies published in English were considered eligible. No explicit date restrictions were applied, and the search identified studies published between 1990 and 2025. Studies from the 1990s were included since this period represented the emergence of a consistent body of early publications evaluating rectal and oral sodium butyrate in ulcerative colitis (and therefore providing historical and mechanistic foundations for the field). Their inclusion was primarily aimed at providing a comprehensive synthesis of the evidence across the entire development of this kind of therapeutic approach.

### 2.2. Eligibility Criteria

Eligibility was defined using the PICO framework:

- Population (P): patients diagnosed with ulcerative colitis, including both adult and pediatric populations;
- Intervention (I): sodium butyrate administration;
- Comparator (C): placebo, standard therapy, or no comparator;
- Outcomes (O): clinical outcomes, including remission, response, efficacy, or improvement.

#### 2.2.1. Inclusion Criteria

Studies were considered eligible if they fulfilled the following criteria:

- Original preclinical or clinical studies investigating sodium butyrate or butyrate-containing formulations administered orally or rectally;
- Human studies involving patients diagnosed with ulcerative colitis or inflammatory bowel disease, or preclinical studies using mammalian models of ulcerative colitis, colitis, or diversion colitis;
- Studies reporting at least one clinically or mechanistically relevant outcome, including:
  ○ clinical disease activity,
  ○ remission rates,
  ○ endoscopic or histological scores,
  ○ inflammatory biomarkers,
  ○ epithelial barrier integrity,
  ○ microbiota alterations,
  ○ neuronal or mechanistic outcomes.

#### 2.2.2. Exclusion Criteria

The following studies were excluded:

- Narrative reviews, systematic reviews, meta-analyses, editorials, or conference abstracts without original primary data;
- Studies investigating non-IBD conditions or non-colonic disease models;
- Studies evaluating short-chain fatty acids without a specific sodium butyrate treatment arm;
- Non-English publications.

### 2.3. Study Selection

A total of 3689 records were identified: 3658 from three bibliographic databases (PubMed/MEDLINE, *n* = 1235; Web of Science, *n* = 917; Scopus, *n* = 1506) and 31 from two trial registries (ClinicalTrials.gov, *n* = 24; WHO ICTRP, *n* = 7).

All records were imported into the Rayyan systematic-review platform (Rayyan Systems Inc., Cambridge, MA, USA). After removal of 1414 duplicate and overlapping records, 2275 records were screened independently and in duplicate by two reviewers (G.I.G. and R.A.I.) in blind mode, of which 2197 were excluded at the title and abstract stage; disagreements were resolved by discussion and, where necessary, by a third reviewer (A.C.).

**Figure 1:**
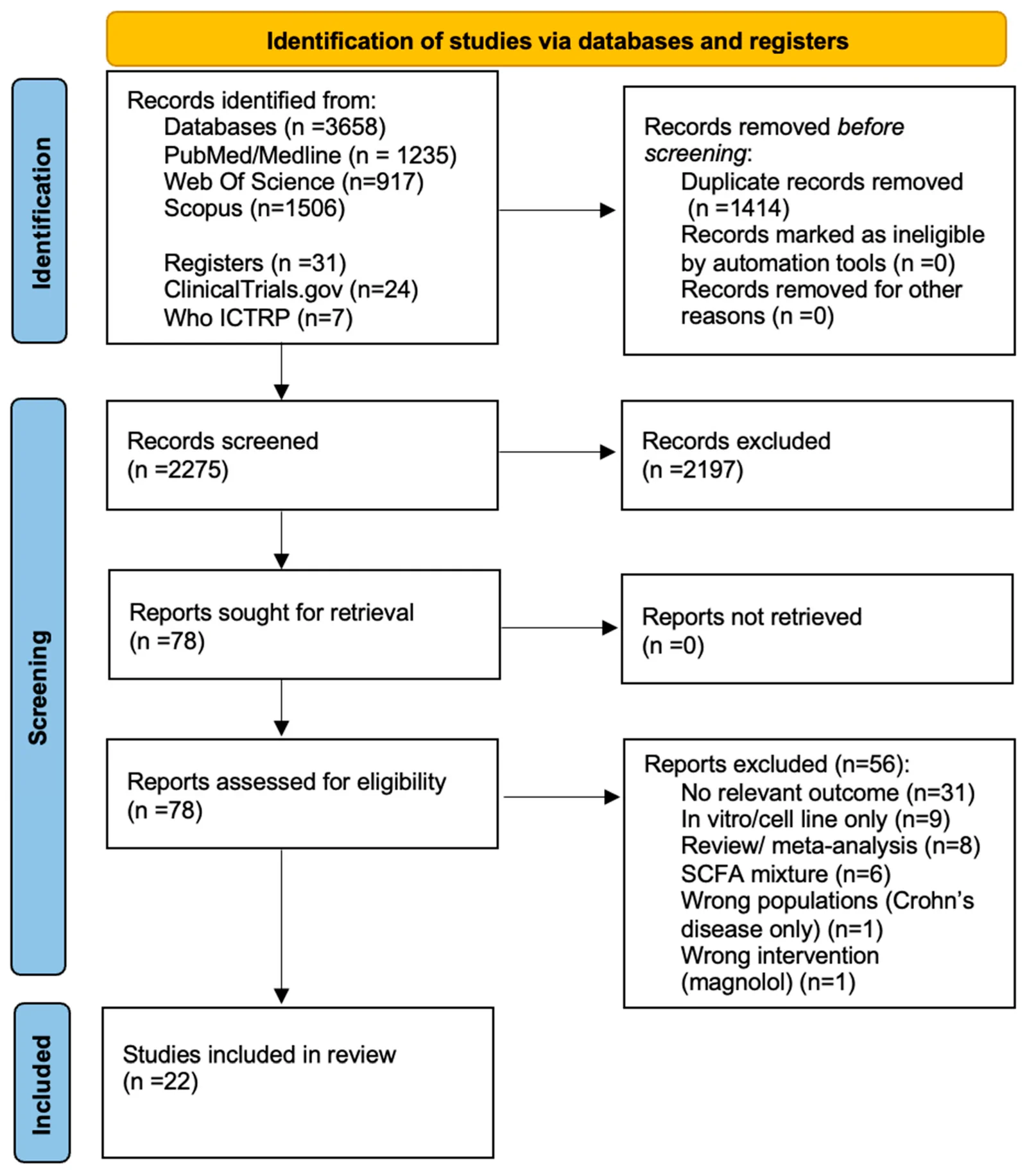
PRISMA flow diagram illustrating the study selection process, including identification, screening, eligibility assessment, and final inclusion of studies.

Seventy-eight full-text reports were sought, retrieved, and assessed for eligibility, of which 56 were excluded, with reasons detailed in Figure 1. Twenty-two studies met the inclusion criteria.

Of the 31 records identified from trial registries, 27 were assessed after removal of overlapping entries, and none yielded includable primary data beyond studies already identified through database searching. The study-selection process is summarized in the PRISMA 2020 flow diagram (Figure 1).

### 2.4. Data Extraction

Data extraction was independently performed by two reviewers using a standardized approach. The following variables were collected from each eligible study:

- author and year of publication;
- study design;
- study population and sample size or experimental model;
- disease subtype;
- intervention characteristics (dose, formulation, route of administration and treatment duration);
- evaluated clinical, biochemical, histological, microbiological, or mechanistic outcomes;
- principal findings and study conclusions.

Discrepancies were resolved by consensus. Where a single trial was reported across more than one publication, it was treated as a single study.

### 2.5. Data Synthesis

Due to substantial heterogeneity among the included studies regarding patient populations, study design, sodium butyrate formulations, routes of administration, treatment duration, and evaluated outcomes, a quantitative meta-analysis was not considered appropriate.

Therefore, a qualitative narrative synthesis was performed.

The included studies were categorized into four predefined thematic sections:

- Clinical studies on adult ulcerative colitis;
- Clinical studies on pediatric inflammatory bowel disease;
- Rectal sodium butyrate therapy in ulcerative colitis;
- Preclinical and mechanistic evidence.

This classification allowed a structured evaluation of the available evidence according to population characteristics, route of administration, and mechanistic focus. The overall certainty of the evidence was appraised narratively, considering study design, methodological rigor, consistency of findings, and translational relevance.

### 2.6. Risk of Bias and Quality Assessment

The risk of bias of the included studies was evaluated using study design-specific tools. Randomized clinical trials were assessed using the revised Cochrane Risk of Bias tool for randomized trials, RoB 2 [13]. The prospective observational study was assessed using ROBINS-I [14]. Preclinical animal studies were evaluated using the SYRCLE risk-of-bias tool [15].

The assessment was performed independently by two reviewers, and disagreements were resolved by discussion. The main domains considered included randomization, allocation concealment, blinding, missing outcome data, outcome measurement, selective reporting, and other potential sources of bias.

No overall numerical quality score was calculated, as risk of bias was assessed by individual methodological domains rather than by a cumulative scoring system. The results of the assessment were used to support the narrative synthesis and to interpret the reliability of the evidence, but they were not used as exclusion criteria.

Due to the heterogeneity of the included studies, the risk-of-bias findings were summarized descriptively and presented in tabular form.

## 3. Results

### 3.1. Risk of Bias Assessment

The risk of bias assessment for all included studies is presented in Table 2. Overall, the methodological quality varied across studies, with differences mainly related to randomization procedures, blinding, allocation concealment, and reporting of outcome data. Most preclinical studies showed some concerns due to limited reporting of methodological details, whereas the clinical studies generally demonstrated a lower risk of bias.

Overall, the methodological quality of the included studies was heterogeneous. Most randomized clinical trials were judged as having low risk of bias or some concerns. The main reasons for some concerns were small sample size, pilot design, short follow-up, single-center design, or incomplete reporting of allocation and blinding procedures.

The prospective observational study by Vernero et al. [16] was judged as having moderate risk of bias, mainly due to its non-randomized design and the possibility of residual confounding.

In preclinical studies, the overall risk of bias was generally judged as some concerns, mainly because several SYRCLE domains, including randomization, allocation concealment, random housing, and blinded outcome assessment, were not consistently reported. No study was excluded based on risk-of-bias assessment.

### 3.2. Clinical Studies in Adult UC

Table 3 summarizes the clinical studies evaluating sodium butyrate in adult patients with ulcerative colitis. The identified studies comprise randomized controlled trials and prospective observational designs, assessing outcomes such as clinical remission, disease activity scores, and biochemical inflammatory markers (e.g., fecal calprotectin and CRP). Globally, the clinical evidence suggests heterogeneous results, ranging from significant improvement in disease activity and remission rates to studies reporting no additional benefit compared to standard therapy.

Vernero et al. [16] conducted a prospective observational study evaluating oral microencapsulated sodium butyrate as add-on therapy in 42 patients with ulcerative colitis in clinical remission, of whom 39 completed 12 months of follow-up. Patients received either sodium butyrate plus mesalamine (*n* = 18) or mesalamine alone (*n* = 21). Therapeutic success (quantified by partial Mayo score ≤ 2 and fecal calprotectin <250 µg/g at 12 months) was significantly higher in the sodium butyrate group (83.3% vs. 47.6%, *p* = 0.022), with greater improvement in alleviating symptoms and a significant reduction in fecal calprotectin levels compared to controls [16]. The authors concluded that microencapsulated sodium butyrate is a beneficial adjunctive therapy for maintaining remission in ulcerative colitis, warranting confirmation in randomized controlled trials.

A recent multicenter double-blind randomized trial conducted by Karłowicz et al. [17] evaluating microencapsulated sodium butyrate (MSB) at doses around 600 mg/day demonstrated improved clinical and endoscopic outcomes in comparison with placebo in patients with a diagnosis of mild-to-moderate UC. This study reported higher rates of clinical remission and biochemical response, along with increased fecal butyrate levels, which were positively correlated with therapeutic response, supporting a potential mechanistic link between luminal SCFA restoration and disease control [17].

Likewise, a series of randomized controlled trials by Firoozi et al. [18,19] evaluating high-dose oral sodium butyrate (600 mg/day) in active UC showed significant reductions in partial Mayo scores and systemic inflammatory markers like ESR and neutrophil-to-lymphocyte ratio. The first study demonstrated improvements in terms of patient-reported outcomes (including anxiety, depression scores, and global health status), suggesting that sodium butyrate may exert broader systemic effects beyond intestinal inflammation [18]. The other trial further showed modulation of circadian rhythm gene expression (CLOCK, BMAL1, PER1/2, CRY1/2), together with improved sleep quality and quality of life indices. The authors suggested a potential gut–brain–immune axis involvement in its therapeutic effects [19].

However, not all studies have shown clear superiority over standard therapy in terms of outcomes. An earlier randomized pilot trial conducted by Vernia et al. assessed oral sodium butyrate (4 g/day) in combination with mesalamine in comparison with mesalamine monotherapy. The trial failed to show any statistically significant differences, despite trends toward improved UC disease activity indices. The absence of significance was primarily attributed to several factors, such as small sample size, short follow-up period and heterogeneity in response measures [20].

Collectively, adult studies suggest that while oral sodium butyrate demonstrates consistent biological activity and potential clinical benefit, the magnitude of its effect is variable and appears highly dependent on formulation, dosing strategy, and study design.

### 3.3. Clinical Studies in Pediatric IBD

The pediatric evidence base consists primarily of randomized, placebo-controlled trials evaluating sodium butyrate as an adjunct to conventional therapy in newly diagnosed inflammatory bowel disease, including both ulcerative colitis and Crohn’s disease (Table 4).

Pietrzak et al. [21] conducted a prospective, randomized, placebo-controlled multicenter trial to evaluate the efficacy of oral sodium butyrate as an adjunct to standard therapy in children and adolescents (aged 6–18 years) with newly diagnosed inflammatory bowel disease (Crohn’s disease or ulcerative colitis). Patients received either sodium butyrate (150 mg twice daily) or placebo for 12 weeks. The study assessed disease activity and fecal calprotectin levels as primary outcomes. Although most patients in both groups achieved remission, no significant differences were observed between the butyrate and placebo groups in remission rates, disease activity, or inflammatory markers [21]. The authors concluded that sodium butyrate supplementation did not provide additional clinical benefit in this population.

Goldiș et al. [22] conducted a unicentric, prospective, randomized, placebo-controlled trial to evaluate the efficacy of oral sodium butyrate as an adjunct to conventional therapy in children and adolescents (aged 7–18 years) with newly diagnosed inflammatory bowel disease. Patients received either 150 mg sodium butyrate once daily or placebo for 12 weeks. The study assessed disease activity, C-reactive protein, and fecal calprotectin as primary outcomes. A significantly higher remission rate was observed in the sodium butyrate group compared to placebo, along with greater reductions in disease activity and inflammatory markers. No adverse events were reported. The authors concluded that sodium butyrate supplementation may be an effective adjunctive treatment in pediatric IBD.

The discrepancy between these two studies may be explained by differences in formulation (standard sodium butyrate vs. microencapsulated delivery systems), dosing schedules, study setting (multicenter vs. unicenter design), and potential differences in baseline disease severity or treatment adherence. Overall, pediatric data remain inconsistent, limiting definitive conclusions regarding efficacy in this population.

### 3.4. Rectal Sodium Butyrate Therapy in UC

Rectal administration of sodium butyrate has been evaluated primarily in distal ulcerative colitis and represents an early therapeutic approach with the objective of delivering high local concentrations directly to the inflamed mucosa (Table 5).

Initial proof-of-concept studies showed strong biological effects. A crossover pilot trial conducted by Scheppach et al. [23] enrolled patients who were diagnosed with distal UC and showed that sodium butyrate enemas significantly reduced stool frequency and rectal bleeding, with improvements in both endoscopic and histologic scores. Additionally, mucosal proliferation indices were also reduced, emphasizing a direct effect on epithelial turnover and repair mechanisms of the mucosa [23].

Scheppach et al. conducted another study [24], designed as a randomized, placebo-controlled trial evaluating topical short-chain fatty acid and butyrate enemas in patients diagnosed with active distal ulcerative colitis. Even though all treatment groups showed improvements regarding disease activity, no significant differences were observed in clinical or histological outcomes in comparison with placebo. However, patients receiving butyrate enemas had fewer endoscopically affected colonic segments after 8 weeks, highlighting a potential beneficial effect [24]. The authors concluded that while butyrate therapy showed promising trends, larger studies were needed to confirm its efficacy.

However, another randomized controlled trial yielded less consistent results in comparison with the previous ones. In a placebo-controlled study by Steinhart et al., nightly sodium butyrate enemas did not show superiority over saline with respect to clinical remission or endoscopic improvement, as similar remission rates were observed in both groups [25].

Hamer et al. [26] evaluated the effects of daily rectal sodium butyrate enemas (100 mM) administered for 20 days in 35 patients with ulcerative colitis in clinical remission. Overall, butyrate produced only modest effects on mucosal inflammation and oxidative stress compared with placebo. A significant increase in the colonic IL-10/IL-12 ratio and higher colonic CCL5 concentrations were observed following treatment, while no consistent improvements were registered in other inflammatory markers, oxidative stress parameters, fecal calprotectin, or CRP [26]. The authors concluded that rectal butyrate enemas had limited anti-inflammatory and antioxidant effects in patients with quiescent ulcerative colitis.

On the whole, rectal butyrate showed clear biological activity at the mucosal level, but clinical efficacy remains inconsistent across several studies (likely influenced by dose, formulation, disease chronicity, and study design).

### 3.5. Preclinical and Mechanistic Evidence

Table 6 summarizes the preclinical (experimental and mechanistic) studies investigating the effects of sodium butyrate in animal models of colitis. These studies employed DSS- and TNBS-induced colitis models, diversion colitis models, and complementary in vitro mechanistic experiments. The studies focused on inflammatory signaling pathways, oxidative stress, epithelial barrier integrity, and gut microbiota modulation. Overall, experimental findings consistently demonstrate anti-inflammatory effects of sodium butyrate, with improvements in colitis severity, mucosal integrity, and microbial composition across different mechanistic pathways.

In an experimental DSS-induced acute colitis model, Vieira et al. [27] evaluated the effects of dietary sodium butyrate supplementation on intestinal inflammation and mucosal injury. Mice receiving butyrate demonstrated a significant attenuation of colonic damage, reflected by reduced histological lesions and improved mucosal architecture compared to untreated DSS controls. In addition, butyrate supplementation was linked to a marked reduction in leukocyte infiltration and a partial normalization of intestinal immune responses, including modulation of cytokine expression patterns. The study supports a protective role of sodium butyrate in acute inflammatory injury, highlighting its capacity to reduce epithelial damage and dampen mucosal immune activation in experimental colitis.

In a DSS-induced colitis model, Li et al. [28] further explored the molecular mechanisms underlying sodium butyrate-mediated protection, focusing on autophagy and intracellular signaling pathways. Their findings demonstrated that butyrate significantly improved clinical disease severity and histopathological scores in a dose-dependent manner. Regarding the mechanism of action, sodium butyrate inhibited activation of the PI3K/AKT/mTOR signaling pathway while simultaneously enhancing autophagy-related protein expression (including LC3-II and Beclin-1). These changes were also responsible for a reduction in inflammatory cytokine levels and restoration of epithelial homeostasis. Moreover, butyrate induced favorable shifts in gut microbiota composition, indicating a combined immunometabolic and microbiome-mediated effect in DSS-induced colitis.

Liang et al. [29] investigated the role of sodium butyrate in epithelial–immune crosstalk using DSS-induced colitis models and complementary in vitro macrophage–goblet cell co-culture systems. The study demonstrated that butyrate promotes polarization of macrophages toward an anti-inflammatory M2 phenotype, characterized by increased CD206 and Arg1 expression. This macrophage shift was associated with enhanced goblet cell differentiation and increased mucin production, particularly MUC2 and SPDEF expression. Mechanistically, these effects were mediated through activation of WNT/ERK signaling pathways and required macrophage-dependent modulation of epithelial responses. Collectively, the findings indicate that sodium butyrate contributes to restoration of the mucus barrier through coordinated immune and epithelial regulation.

Fan et al. [30] explored the mechanistic effects of sodium butyrate in an experimental model of dextran sodium sulfate (DSS)-induced colitis, demonstrating that its protective role is closely linked to the inhibition of mitochondrial reactive oxygen species (ROS)-dependent macrophage pyroptosis. In vivo, sodium butyrate attenuated colonic inflammation and reduced circulating and tissue inflammatory mediators. Complementary in vitro experiments showed suppression of pro-inflammatory signaling pathways (including ERK and NF-κB) and decreased macrophage pyroptotic activity through preservation of mitochondrial function and reduction of oxidative stress. The authors also reported improved epithelial barrier integrity secondary to modulation of macrophage-mediated inflammation. These findings suggest that sodium butyrate may exert therapeutic effects in colitis by targeting mitochondrial dysfunction and inflammatory cell death pathways.

Caetano et al. [31] investigated the effects of sodium butyrate on the enteric nervous system in a TNBS-induced colitis mouse model, focusing on neuronal integrity and glial responses. Their findings demonstrated that experimental colitis was associated with significant loss of enteric neurons and increased glial activation, reflecting disruption of intestinal neuromuscular homeostasis. Sodium butyrate treatment attenuated these changes by preserving neuronal populations, including nNOS- and ChAT-positive neurons, and reducing reactive gliosis as indicated by decreased GFAP expression. Additionally, butyrate modulated GPR41-related signaling pathways, which are implicated in gut microbial metabolite sensing and neuroimmune regulation. Overall, the study highlights a neuroprotective role of sodium butyrate in colitis, extending its effects beyond epithelial and immune mechanisms to the enteric nervous system.

Pacheco et al. [32] investigated the effects of sodium butyrate enemas in a rat model of diversion colitis, comparing them with glutamine and saline controls. Butyrate treatment resulted in significant improvement of endoscopic and histological inflammatory scores, accompanied by restoration of normal mucosal architecture. The intervention also normalized goblet cell density and reduced epithelial apoptosis. At the molecular level, butyrate significantly decreased pro-inflammatory cytokines, including IL-1β and TNF-α, while modulating TGF-β expression. These findings indicate that butyrate exerts trophic, anti-inflammatory, and anti-fibrotic effects in colonic mucosa deprived of fecal stream stimulation.

Fan et al. [33] evaluated a butyrate-based polymer nanoplatform designed to improve mucosal delivery and therapeutic efficacy in experimental IBD models. The engineered system demonstrated enhanced intestinal targeting and prolonged retention, resulting in more effective attenuation of oxidative stress and inflammatory responses compared to conventional approaches. Treatment significantly reduced pro-inflammatory cytokine expression and improved epithelial regeneration and wound healing capacity. Transcriptomic analysis further revealed regulation of lipid metabolism and inflammatory signaling pathways, while microbiota profiling showed restoration of microbial balance with enrichment of beneficial taxa. These findings suggest that optimized delivery systems may enhance the therapeutic potential of butyrate in intestinal inflammation.

Jourova et al. [34] evaluated the effects of sodium butyrate in a DSS-induced murine model of ulcerative colitis, with particular focus on the gut–liver axis and hepatic drug metabolism. In addition to confirming that butyrate pretreatment attenuated colonic inflammation and improved epithelial barrier integrity (partly through restoration of tight junction protein ZO-1), the authors investigated its impact on hepatic cytochrome P450 (CYP) enzymes. They demonstrated that both DSS-induced inflammation and butyrate administration modulated the expression and activity of selected CYP isoforms, with CYP3A enzymes appearing particularly sensitive to these interventions [34]. The study highlights that microbiota-derived metabolites such as butyrate may influence not only intestinal inflammation but also hepatic drug-metabolizing capacity, suggesting potential implications for pharmacotherapy in patients with inflammatory bowel disease.

In their experimental study, Dou et al. [35] investigated the protective effects of dietary sodium butyrate (NaB) in a DSS-induced colitis mouse model. Male C57BL/6 mice were randomized into control, DSS, and NaB + DSS groups, with NaB administered orally before and during colitis induction. Disease severity was assessed using the Disease Activity Index (DAI), colon length, spleen weight, and histological scoring. NaB supplementation significantly attenuated clinical and histological signs of colitis, including reduced DAI scores, preservation of colon length, decreased splenomegaly, and diminished inflammatory cell infiltration. Furthermore, 16S rRNA sequencing of cecal contents demonstrated that NaB increased α-diversity and favorably modulated β-diversity compared to DSS alone. At the phylum level, NaB partially restored DSS-induced dysbiosis, notably increasing the Firmicutes/Bacteroidetes ratio and reducing Proteobacteria abundance. Linear Discriminant Analysis Effect Size (LEfSe analysis) was performed and confirmed distinct microbial signatures among groups [35]. Collectively, the findings suggest that sodium butyrate mitigates experimental colitis through both anti-inflammatory effects and modulation of gut microbiota composition.

Butzner et al. [36] evaluated the effects of daily sodium butyrate enemas in a rat model of TNBS-induced colitis. In comparison with saline or no treatment groups, butyrate therapy improved clinical recovery by resolving diarrhea, alleviating colonic damage and myeloperoxidase activity, and restoring short-chain fatty acid-stimulated sodium absorption [36]. The authors stated that butyrate enemas promote mucosal healing and reduce intestinal inflammation in experimental colitis.

Liu et al. [37] assessed the effects of oral sodium butyrate in a DSS-induced mouse model of colitis. Sodium butyrate significantly decreased disease severity by reducing weight loss, disease activity index, inflammatory cytokine expression, and oxidative stress. Moreover, antioxidant activity was increased. Mechanistically, butyrate inhibited ferroptosis, enhanced ERK/STAT3 signaling, and favorably regulated the gut microbiota [37]. The authors suggested that these pathways contribute to its protective effects in experimental inflammatory bowel disease.

Collectively, preclinical data converge on a multi-target mechanism involving immune modulation, epithelial barrier restoration, metabolic regulation, and microbiota restructuring, supporting the biological plausibility of butyrate as a therapeutic adjunct in ulcerative colitis.

## 4. Discussion

### 4.1. Clinical and Practical Perspectives

From a practical standpoint, the most consistent clinical signal is attributed to remission-related outcomes and biochemical inflammatory markers. In adult populations, the adjunctive administration of sodium butyrate alongside mesalamine was linked to improved maintenance of remission and decreased fecal calprotectin levels, showing a possible benefit in patients with clinically stable disease (who require long-term control) [16]. This finding is particularly relevant for real-world settings, where prevention of relapse and reduction of subclinical inflammation are the main therapeutic goals.

In contrast, the available pediatric data are less consistent. One of the included randomized placebo-controlled trials did not demonstrate significant advantage of sodium butyrate over standard therapy in terms of remission or inflammatory markers [21], underscoring that its benefit might not be global across age groups or disease phenotypes. However, the other randomized study (which is more recent) demonstrated improved remission rates and reductions in fecal calprotectin and CRP in the group with butyrate supplementation, suggesting that clinical efficacy could depend on several factors like formulation, dosing regimen, or baseline disease activity [22]. These discrepancies are of the utmost importance from a clinical decision-making perspective because they indicate that patient selection can be critical when considering supplementation.

The heterogeneity of the available clinical evidence is perhaps multifactorial. Differences regarding pharmaceutical formulation, oral versus topical administration, drug release characteristics, duration of treatment, disease extent and activity, concomitant therapies and patient selection might have contributed to the variability of clinical outcomes. A further and largely unaccounted source of variability is the gut microbiota itself. Exogenous butyrate is administered against a highly variable endogenous background: patients with ulcerative colitis characteristically show depletion of butyrate-producing bacteria, but the magnitude of this depletion differs substantially between individuals and disease states. Prior or concomitant exposure to antibiotics, probiotics and prebiotics represents an additional, largely uncontrolled modifier of community composition. Particularly, microencapsulated oral formulations could provide more sustained colonic delivery than conventional preparations, while rectal enemas are limited by patient adherence, retention time and disease distribution. All these methodological differences complicate direct comparisons across studies and currently preclude identification of an optimal therapeutic regimen.

From a broader clinical perspective, preclinical evidence consistently demonstrates anti-inflammatory effects and improvement of mucosal integrity; however, all these findings primarily support biological plausibility rather than direct clinical decision-making. Thus, their relevance is centrally supportive in the understanding process of the mechanisms behind observed clinical effects rather than guiding therapeutic use.

The available evidence indicates that sodium butyrate might have a potential adjunctive role in the clinical management of UC, particularly in the context of maintenance therapy and modulating the inflammatory activity of the disease. However, we must acknowledge that the overall clinical utility remains limited due to heterogeneous results across studies and lack of standardized therapeutic protocols.

### 4.2. Preclinical Perspectives

Preclinical studies consistently indicate that sodium butyrate exerts robust anti-inflammatory and barrier-protective effects in experimental models of colitis, predominantly in DSS-induced systems. Even though these findings cannot be directly translated into clinical recommendations, they provide important supportive evidence for the biological plausibility of its potential benefits in UC.

From a translational point of view, the most relevant observation across the included preclinical studies is the consistent reduction in disease severity indices, including improved Disease Activity Index (DAI), preservation of colon length, and decreased histological damage. These outcomes demonstrate a global attenuation of inflammatory injury rather than isolated pathway-specific effects, implying a broad protective profile in chemically induced colitis models [34,35].

Notably, beyond the improvement of macroscopic disease, butyrate supplementation is repeatedly linked to restoration of epithelial integrity, particularly via increased expression of tight junction proteins such as ZO-1 and improved mucosal barrier function [34]. This is clinically relevant considering that epithelial barrier failure is a central feature of disease pathophysiology, and interventions targeting the restoration of the barrier are considered a key therapeutic strategy in inflammatory bowel disease.

Additionally, experimental findings indicate that sodium butyrate might exert its effects through the modulation of the inflammatory response at the cellular level, including suppression of macrophage-driven inflammation and decrease of oxidative stress-related injury. For instance, inhibition of macrophage pyroptosis and reduction of mitochondrial reactive oxygen species constituted potential mechanisms associated with decreased inflammatory signaling in colitis models [30]. While these mechanistic insights are not directly actionable in clinical practice, they help explain the consistent anti-inflammatory phenotype observed across the models.

Another relevant experimental observation, even though not the aim of our analysis, is related to the impact on gut microbiota composition, with partial restoration of dysbiosis and improvement in microbial diversity indices following butyrate administration [35]. Even if microbiota changes are complex and sometimes difficult to interpret in isolation, these findings support the concept that butyrate might act not only as a metabolic substrate but also as a modulator of host–microbe interactions, which are central in disease pathogenesis.

Finally, one study suggests that the effects of butyrate could extend beyond the gut, influencing systemic pathways such as the gut–liver axis and hepatic drug metabolism [34]. While these observations are intriguing, their direct clinical relevance remains uncertain and does not imply therapeutic decision-making in the present.

### 4.3. Key Claims and Evidence Levels

Table 7 summarizes the main evidence-derived claims regarding sodium butyrate in ulcerative colitis and inflammatory bowel disease, stratified by study type and strength of evidence. Oral microencapsulated sodium butyrate shows the most consistent benefit as an adjunct therapy in adult UC, improving clinical remission, disease activity indices and inflammatory biomarkers across multiple randomized controlled trials. Oral supplementation in active UC also demonstrates additional effects on psychological symptoms and quality of life. In pediatric populations, results are heterogeneous, with conflicting evidence depending on formulation, dosing and study design. Rectal butyrate enemas show variable efficacy, varying from beneficial effects in small mechanistic or pilot studies to no superiority over placebo in larger randomized trials. Preclinical studies consistently support anti-inflammatory, barrier-protective, immunomodulatory and microbiota-modulating effects of sodium butyrate across DSS and TNBS models, including mechanisms involving autophagy, macrophage polarization and epithelial repair. On the whole, oral butyrate represents a promising adjunctive therapy, while rectal administration shows inconsistent clinical benefit.

### 4.4. Limitations of the Current Study

This systematic review has several limitations that must be acknowledged when interpreting the results.

First, the number of eligible studies was limited, with only a small number of clinical and preclinical studies meeting the predefined inclusion criteria. This reflects the scarcity of high-quality evidence specifically evaluating sodium butyrate in ulcerative colitis and limits the strength of the conclusions that can be drawn.

Second, substantial heterogeneity was observed across the included studies. Differences regarding study design (randomized controlled trials, observational studies, and animal experiments), patient populations (adult versus pediatric, active versus quiescent disease), and experimental models did not allow for direct comparisons between the results and made quantitative synthesis via meta-analysis infeasible.

Third, variability in intervention protocols represents another important limitation. The dose, formulation, route of administration, and duration of sodium butyrate supplementation differed considerably between studies, making it difficult to find an optimal therapeutic regimen and to standardize its clinical application.

Fourth, outcome measures were inconsistent across the selected studies. Clinical endpoints like remission, disease activity scores, and biochemical markers (e.g., fecal calprotectin, CRP) were not uniformly reported, limiting the ability to perform analyses and directly compare efficacy outcomes.

Fifth, the inclusion of both human and animal studies introduces translational limitations. While experimental models provide valuable insights on mechanisms, particularly DSS-induced colitis models, they do not fully replicate the environment of human disease, which limits direct clinical applicability.

Sixth, several included studies are subject to methodological limitations, including small sample sizes, observational designs, and potential confounding factors. In some cases, incomplete reporting of allocation methods introduces the risk of bias and affects the reliability of reported outcomes.

Finally, even though a comprehensive search strategy was applied across multiple databases, there remains a possibility of publication bias, as studies with negative or inconclusive results could be underrepresented in the available literature.

Overall, the available evidence supports a therapeutic role for sodium butyrate in ulcerative colitis, but firm conclusions are limited by heterogeneous formulations, inconsistent dosing regimens and a scarcity of delivery systems optimized for distal disease. The low bioavailability and poor palatability of oral preparations suggest that locally targeted, topical approaches may offer greater benefit in distal colitis and warrant further investigation. Additional well-designed studies are needed to standardize dosing, compare formulation strategies, and develop delivery systems capable of sustained mucosal retention and local release in the distal colon. Rigorous preclinical evaluation, assessing inflammatory activity, oxidative stress and epithelial barrier integrity, followed by controlled clinical trials, will be essential to define the true therapeutic value of sodium butyrate and to translate these findings into clinical practice.

## 5. Conclusions

The current literature suggests that sodium butyrate has a potentially beneficial role as an adjunctive approach in the management of UC, mostly because of its anti-inflammatory properties and its capacity to ensure intestinal barrier integrity. Clinical studies report encouraging results regarding the maintenance of remission and decrease of inflammatory activity. However, the available evidence remains limited and heterogeneous (with inconsistent findings across different study designs and populations). Consequently, the available data do not support definitive conclusions regarding its routine clinical use.

At the same time, preclinical studies consistently demonstrate the protective effects of sodium butyrate in experimental colitis models, including a decrease in intestinal inflammation, improvement of epithelial integrity, modulation of oxidative stress, and partial restoration of balance regarding gut microbiota. These findings reinforce the biological plausibility of sodium butyrate as a therapeutic adjunct in UC. Even more, mechanistic insights into the clinical observations reported so far are provided.

All in all, sodium butyrate represents a promising investigational adjunctive therapy. However, future well-designed, large-scale randomized controlled trials with standardized formulations, dosing regimens, and clearly defined patient populations are mandatory in order to better establish its clinical efficacy and draw conclusions on the therapeutic applicability.

## Figures and Tables

**Table 1:** Electronic search strategies used for the bibliographic databases and clinical trial registries included in the systematic review.

Database/Registry	Search Strategy
PubMed/MEDLINE	(“Colitis, Ulcerative”[Mesh] OR “Proctocolitis”[Mesh] OR “ulcerative colitis”[tiab] OR “inflammatory bowel disease”[tiab] OR IBD[tiab] OR colitis[tiab] OR proctitis[tiab] OR proctosigmoiditis[tiab]) AND (“Butyrates”[Mesh] OR “Butyric Acid”[Mesh] OR butyrate[tiab] OR “butyric acid”[tiab])
Scopus	TITLE-ABS-KEY(“ulcerative colitis” OR “inflammatory bowel disease” OR IBD OR colitis OR proctitis OR proctosigmoiditis) AND TITLE-ABS-KEY(butyrate* OR “butyric acid”)
Web of Science (Core Collection)	TS = (“ulcerative colitis” OR “inflammatory bowel disease” OR colitis OR proctitis OR proctosigmoiditis) AND TS = (butyrate* OR “butyric acid”)
ClinicalTrials.gov	“Ulcerative colitis” AND butyrate
WHO International Clinical Trials Registry Platform (ICTRP)	“Ulcerative colitis” AND butyrate

**Table 2:** Risk of bias assessment of the included studies.

Study	Study Design	Tool Used	Overall Risk of Bias	Main Reason
Vernero et al., 2020 [16]	Prospective observational study	ROBINS-I	Moderate	Non-randomized treatment allocation; possible residual confounding
Karłowicz et al., 2025 [17]	Multicenter double-blind randomized placebo-controlled trial	RoB 2	Low	Randomized, double-blind, placebo-controlled design with clearly defined outcomes
Firoozi et al., 2025 [18]	Double-blind randomized placebo-controlled trial	RoB 2	Low	Randomized double-blind design; clearly reported intervention and comparator
Firoozi et al., 2024 [19]	Double-blind randomized placebo-controlled trial	RoB 2	Low	Randomized double-blind design; clearly reported outcomes and follow-up
Vernia et al., 2000 [20]	Double-blind randomized placebo-controlled pilot trial	RoB 2	Some concerns	Small sample size, pilot design, short follow-up
Pietrzak et al., 2022 [21]	Multicenter randomized placebo-controlled trial	RoB 2	Low	Multicenter randomized placebo-controlled design; adequately defined clinical outcomes
Goldiș et al., 2025 [22]	Unicenter randomized placebo-controlled trial	RoB 2	Some concerns	Single-center design; limited information on allocation concealment/blinding
Scheppach et al., 1992 [23]	Single-blind randomized crossover pilot study	RoB 2 crossover	Some concerns	Very small sample size, single-blind design, possible carryover-related limitations
Scheppach et al., 1996 [24]	Randomized placebo-controlled parallel trial	RoB 2	Some concerns	Small sample size; early discontinuation
Steinhart et al., 1996 [25]	Randomized placebo-controlled trial	RoB 2	Some concerns	Older study with limited reporting of allocation/blinding details
Hamer et al., 2010 [26]	Randomized double-blind placebo-controlled parallel trial	RoB 2	Some concerns	Randomized double-blind placebo-controlled design, but small sample size, short intervention period, and limited clinical outcome
Vieira et al., 2012 [27]	DSS-induced acute colitis animal study	SYRCLE	Some concerns	Randomization and blinded outcome assessment not consistently reported
Li et al., 2025 [28]	DSS-induced colitis animal study	SYRCLE	Some concerns	Preclinical controlled design, but allocation concealment/blinding details unclear
Liang et al., 2022 [29]	Animal and in vitro mechanistic study	SYRCLE	Some concerns	Animal randomization/blinding incompletely reported; in vitro component interpreted descriptively
Fan et al., 2025 [30]	Animal and in vitro mechanistic study	SYRCLE	Some concerns	Allocation concealment and blinded assessment not clearly reported
Caetano et al., 2023 [31]	TNBS-induced colitis animal study	SYRCLE	Some concerns	Randomization/blinding procedures incompletely reported
Pacheco et al., 2012 [32]	Diversion colitis rat model	SYRCLE	Some concerns	Allocation and blinded histological assessment not clearly reported
Fan et al., 2024 [33]	Preclinical IBD model with sodium butyrate-based delivery platform	SYRCLE	Some concerns	Complex animal model; randomization and outcome blinding not fully clear
Jourová et al., 2022 [34]	DSS-induced colitis animal study	SYRCLE	Some concerns	Several SYRCLE domains insufficiently reported
Dou et al., 2020 [35]	DSS-induced colitis animal study	SYRCLE	Some concerns	Randomization, housing, and blinded outcome assessment not consistently reported
Butzner et al., 1996 [36]	TNBS-induced experimental colitis rat study	SYRCLE	Some concerns	Incomplete reporting of allocation concealment, random housing, and blinded outcome assessment
Liu et al., 2025 [37]	DSS-induced IBD mouse study	SYRCLE	Some concerns	Randomization reported; allocation concealment, random housing, and blinding unclear

RoB 2, Cochrane Risk of Bias 2 tool; ROBINS-I, Risk Of Bias In Non-randomized Studies of Interventions; SYRCLE, Systematic Review Center for Laboratory animal Experimentation risk-of-bias tool; DSS, dextran sulfate sodium; TNBS, 2,4,6-trinitrobenzenesulfonic acid; IBD, inflammatory bowel disease.

**Table 3:** Clinical studies evaluating sodium butyrate in adult UC.

Study	Design	Population	Treatment	Outcomes Assessed	Key Results	Conclusion
Vernero et al., 2020 [16]	Prospective observational	UC in remission (*n* = 39)	Sodium butyrate + mesalamine vs. mesalamine	Partial Mayo score, fecal calprotectin	Higher remission maintenance (83.3% vs. 47.6%, *p* = 0.022); decrease of calprotectin; improved residual symptoms	Beneficial adjunct for maintaining remission
Karłowicz et al., 2025 [17]	Multicenter, double-blind, randomized, placebo-controlled trial	Mild-to-moderate UC (*n* = 98 analyzed)	Microencapsulated sodium butyrate 300 mg BID vs. placebo, 8 weeks	Total Mayo Score, clinical/endoscopic/biochemical remission, fecal butyrate	Higher rates of clinical and endoscopic remission; improved biochemical markers; fecal butyrate increased and correlated with response	MSB may improve clinical and mucosal outcomes as add-on therapy
Firoozi et al., 2025 [18]	Double-blind, randomized, placebo-controlled trial	Active UC (*n* = 36, 18/arm)	Sodium butyrate 600 mg/day vs. placebo, 12 weeks	Partial Mayo score, ESR, NLR, PLR, HADS, GHQ	Reduced disease activity and systemic inflammation; improved anxiety, depression, and general health scores	Sodium butyrate improves inflammatory and psychological outcomes in active UC
Firoozi et al., 2024 [19]	Double-blind, randomized, placebo-controlled trial	Active UC (*n* = 36, 18/arm)	Sodium butyrate 600 mg/day vs. placebo, 12 weeks	Circadian gene expression (CLOCK, BMAL1, PER1/2, CRY1/2), PSQI, IBDQ-9, calprotectin, hs-CRP	Modulation of circadian genes; improved sleep quality and QoL; reduced inflammatory markers	Suggests gut–circadian–immune interaction effects
Vernia et al., 2000 [20]	Randomized, double-blind, placebo-controlled pilot trial	Mild-to-moderate UC (*n* = 30, 25 completers)	Sodium butyrate 4 g/day + mesalazine vs. mesalazine + placebo, 6 weeks	UCDAI, clinical, endoscopic, histologic indices	Both groups improved; no statistically significant between-group difference	Limited evidence of additional benefit over mesalazine

UC, ulcerative colitis; BID, twice daily; ESR, erythrocyte sedimentation rate; NLR, neutrophil-to-lymphocyte ratio; PLR, platelet-to-lymphocyte ratio; HADS, Hospital Anxiety and Depression Scale; GHQ, General Health Questionnaire; PSQI, Pittsburgh Sleep Quality Index; IBDQ-9, Inflammatory Bowel Disease Questionnaire-9; hs-CRP, high-sensitivity C-reactive protein; UCDAI, Ulcerative Colitis Disease Activity Index; QoL, quality of life.

**Table 4:** Clinical studies evaluating sodium butyrate in pediatric IBD (UC and Crohn’s disease).

Study	Design	Population	Treatment	Outcomes Assessed	Key Results	Conclusion
Pietrzak et al., 2022 [21]	Multicenter, randomized, double-blind, placebo-controlled trial	Newly diagnosed pediatric IBD (UC and CD), 6–18 years (*n* = 72)	Sodium butyrate 150 mg BID vs. placebo, 12 weeks	PUCAI/PCDAI, clinical remission, fecal calprotectin, CRP	No significant differences in remission rates or inflammatory markers between groups; both groups improved under standard therapy	No additional clinical benefit of sodium butyrate in pediatric IBD
Goldiș et al., 2025 [22]	Unicenter, randomized, placebo-controlled trial	Newly diagnosed pediatric IBD (UC and CD), 7–18 years (*n* = 88)	Microencapsulated sodium butyrate 150 mg once daily vs. placebo, 12 weeks	PUCAI/PCDAI, CRP, fecal calprotectin, clinical remission	Higher remission rates in butyrate group; significant reduction in CRP and fecal calprotectin; no adverse events reported	MSB may be effective as adjunct therapy in pediatric IBD

UC, ulcerative colitis; CD, Crohn’s disease; IBD, inflammatory bowel disease; BID, twice daily; CRP, C-reactive protein; PUCAI, Pediatric Ulcerative Colitis Activity Index; PCDAI, Pediatric Crohn’s Disease Activity Index; MSB, microencapsulated sodium butyrate.

**Table 5:** Rectal sodium butyrate therapy in UC (enema studies).

Study	Design	Population	Treatment	Outcomes Assessed	Key Results	Conclusion
Scheppach et al., 1992 [23]	Single-blind, randomized, crossover pilot study	Distal UC refractory/intolerant to standard therapy (*n* = 10)	Sodium butyrate enemas 100 mmol/L vs. placebo, 2-week periods	Stool frequency, rectal bleeding, endoscopic score, histology, crypt cell proliferation	Marked clinical improvement during butyrate phase: reduced stool frequency and bleeding; improved endoscopic and histologic scores; decreased crypt proliferation	Strong proof-of-concept for local mucosal benefit
Scheppach et al., 1996 [24]	Randomized, placebo-controlled trial	47 patients with active distal UC	SCFA enemas, butyrate enemas (100 mM), or placebo twice daily for 8 weeks	Disease activity index (DAI), endoscopic and histological scores	No significant differences in DAI or histology; butyrate reduced the extent of endoscopically affected colonic segments compared with placebo.	Butyrate showed a trend toward benefit, but no significant overall superiority over placebo.
Steinhart et al., 1996 [25]	Randomized, placebo-controlled trial	Left-sided/distal UC (*n* = 38)	Sodium butyrate enemas 80 mM nightly vs. saline, 6 weeks	Clinical index, endoscopy, histology, remission rates	No significant difference vs. placebo; similar remission rates; no toxicity	No demonstrated clinical superiority over placebo
Hamer et al., 2010 [26]	Randomized, placebo-controlled trial	35 UC patients in clinical remission	100 mM sodium butyrate enema vs. placebo for 20 days	Inflammatory and oxidative stress markers, fecal calprotectin, CRP	Minor effects only; increased IL-10/IL-12 ratio and CCL5, with no significant changes in most other markers.	Butyrate enemas showed limited anti-inflammatory and antioxidant benefits in quiescent UC.

UC, ulcerative colitis; SCFA, short-chain fatty acids; DAI, Disease Activity Index; CRP, C-reactive protein; IL, interleukin; CCL5, C-C motif chemokine ligand 5.

**Table 6:** Experimental and mechanistic studies investigating the effects of sodium butyrate in animal models of colitis.

Study	Model	Intervention	Mechanisms Investigated	Key Findings	Conclusion
Vieira et al., 2012 [27]	DSS-induced acute colitis (mouse)	Oral sodium butyrate (dietary supplementation)	Cytokine profile, immune cell infiltration, mucosal integrity	Reduced colonic inflammation, decreased leukocyte infiltration, improved mucosal lesions, partial normalization of immune responses	Confirms anti-inflammatory and mucosal protective effects in acute colitis
Li et al., 2025 [28]	DSS-induced colitis (mouse)	Oral sodium butyrate (dose-dependent)	PI3K/AKT/mTOR pathway, autophagy, microbiota	Improved clinical and histological severity; increased autophagy markers (LC3, Beclin-1); inhibited PI3K/AKT/mTOR; microbiota shift toward beneficial taxa	Butyrate restores barrier via autophagy and microbiota modulation
Liang et al., 2022 [29]	DSS + in vitro macrophage/goblet cell models	Sodium butyrate	Macrophage polarization, WNT/ERK signaling, mucus barrier	Induced M2 macrophage polarization; increased goblet cells and MUC2 production; enhanced mucus barrier via WNT/ERK axis	Key role in epithelial–immune crosstalk and mucus repair
Fan et al., 2025 [30]	DSS colitis + in vitro macrophages	Sodium butyrate	Mitochondrial ROS, macrophage pyroptosis, NF-κB/ERK	Reduced mitochondrial ROS; inhibited macrophage pyroptosis; suppressed NF-κB and ERK signaling; improved epithelial integrity	Targets mitochondrial dysfunction and inflammatory cell death
Caetano et al., 2023 [31]	TNBS colitis (mouse)	Sodium butyrate	Enteric nervous system, GPR41 signaling	Prevented neuronal loss, reduced glial activation, preserved neuronal markers (nNOS, ChAT); improved enteric structure	Demonstrates neuroprotective effects via GPR41-related pathways
Pacheco et al., 2012 [32]	Diversion colitis (rat)	Butyrate vs. glutamine enemas	Cytokines (IL-1β, TNF-α, TGF-β), histology, apoptosis	Reduced inflammation and fibrosis; restored goblet cells; normalized cytokine levels	Confirms trophic and anti-inflammatory effects in diversion colitis
Fan et al., 2024 [33]	IBD mouse models	Butyrate-based polymer nanoplatform	ROS, transcriptomics, microbiota, epithelial healing	Reduced oxidative stress; improved epithelial migration; normalized lipid metabolism; reshaped microbiota	Enhanced therapeutic efficacy via delivery optimization
Jourová et al., 2022 [34]	DSS colitis (mouse)	Sodium butyrate pretreatment	Gut–liver axis, CYP450 metabolism	Reduced inflammation; modulated ZO-1 expression; altered hepatic CYP3A activity	Extends effects beyond gut to drug metabolism
Dou et al., 2020 [35]	DSS-induced colitis (mouse)	Oral sodium butyrate	Gut microbiota composition (16S rRNA, diversity indices, LEfSe analysis)	Improved disease activity index, preserved colon length, increased microbial diversity, restored Firmicutes/Bacteroidetes ratio, reduced Proteobacteria abundance	Demonstrates that microbiota modulation is a central mechanism of butyrate-mediated protection
Butzner et al., 1996 [36]	TNBS-induced colitis (rat)	Daily 100 mM sodium butyrate enemas	Mucosal repair, inflammation, SCFA-stimulated Na^+^ absorption	Reduced colonic damage and MPO activity, restored Na^+^ absorption, improved clinical recovery.	Butyrate enemas promoted mucosal healing and reduced inflammation in experimental colitis.
Liu et al., 2025 [37]	DSS-induced colitis (mouse)	Oral sodium butyrate (low/high dose) vs. 5-ASA	Ferroptosis, ERK/STAT3 signaling, gut microbiota	Reduced disease severity, inflammation and ferroptosis; increased ERK/STAT3 phosphorylation; improved antioxidant status and gut microbiota composition.	Sodium butyrate alleviated experimental colitis by inhibiting ferroptosis and modulating ERK/STAT3 signaling and the intestinal microbiota.

DSS, dextran sulfate sodium; TNBS, 2,4,6-trinitrobenzenesulfonic acid; IBD, inflammatory bowel disease; SCFA, short-chain fatty acid; ROS, reactive oxygen species; MPO, myeloperoxidase; PI3K, phosphoinositide 3-kinase; AKT, protein kinase B; mTOR, mechanistic target of rapamycin; LC3, microtubule-associated protein 1 light chain 3; ERK, extracellular signal-regulated kinase; STAT3, signal transducer and activator of transcription 3; NF-κB, nuclear factor kappa B; WNT, Wingless/Integrated signaling pathway; MUC2, mucin 2; GPR41, G protein-coupled receptor 41 (FFAR3); nNOS, neuronal nitric oxide synthase; ChAT, choline acetyltransferase; IL-1β, interleukin-1 beta; TNF-α, tumor necrosis factor-alpha; TGF-β, transforming growth factor-beta; ZO-1, zonula occludens-1; CYP3A, cytochrome P450 3A; LEfSe, Linear Discriminant Analysis Effect Size; 5-ASA, 5-aminosalicylic acid; GSH, glutathione; SOD, superoxide dismutase.

**Table 7:** Summary of key claims and supporting evidence on sodium butyrate in ulcerative colitis and IBD.

Claim	Evidence Strength	Reasoning	Papers
Oral microencapsulated sodium butyrate is an effective add-on for inducing remission in mild–moderate adult UC	Strong	Multiple randomized, double-blind, placebo-controlled trials show higher clinical/endoscopic remission and biochemical response vs. placebo with 600 mg/day MSB and good safety	[17,18,19]
Oral sodium butyrate plus mesalazine improves disease activity more than mesalazine alone in UC	Moderate	One randomized, double-blind pilot trial (4 g/day, 6 weeks) showed greater UCDAI and clinical score improvement vs. mesalazine alone, though between-group remission rates were similar and sample was small	[20]
Sodium butyrate enemas improve refractory distal UC	Moderate	Small single-blind crossover trial (100 mM enemas, 2 weeks) in distal UC unresponsive/intolerant to standard therapy showed reduced stool frequency, bleeding, and endoscopic/histologic activity vs. placebo; no large confirmatory trial	[23]
Nightly butyrate enemas at 80 mM are not superior to placebo in distal UC	Moderate	Randomized controlled trial (38 patients) found similar rates of clinical improvement and remission between butyrate and saline arms after 6 weeks	[25]
Oral sodium butyrate reduces disease activity, inflammatory markers, and psychological symptoms in active UC	Moderate	Two double-blind RCTs (600 mg/day, 12 weeks) demonstrated reduced ESR, NLR, partial Mayo score, calprotectin, hs-CRP and improved anxiety, depression, sleep and QoL vs. placebo	[18,19]
Pediatric low-dose sodium butyrate (150 mg 1–2×/day) as add-on has inconsistent benefit in new IBD	Moderate	One multicenter RCT (twice-daily) showed no difference in remission or calprotectin at 12 weeks, whereas a unicentric RCT (once-daily MSB) reported higher remission and larger CRP and calprotectin reductions vs. placebo	[21,22]
Sodium butyrate attenuates DSS- or TNBS-induced colitis in experimental models	Strong	Several controlled animal studies show reduced inflammation, leukocyte infiltration, improved trophism, mucosal healing, and normalized lymphoid profiles with butyrate	[27,28,36]
Butyrate or glutamine enemas reduce inflammation and fibrosis in diversion colitis	Moderate	Diversion colitis model treated with butyrate or glutamine enemas showed lower endoscopic/histologic scores, normalized collagen, goblet cells, apoptosis, and cytokines vs. saline	[32]
Butyrate protects enteric neurons and modulates glia in TNBS colitis	Moderate	Mouse TNBS model: 100 mg/kg sodium butyrate reduced loss of nNOS- and ChAT-positive myenteric neurons, limited glial proliferation, and attenuated morphological damage vs. untreated colitis	[31]
Butyrate alleviates DSS colitis via autophagy and PI3K-AKT-mTOR inhibition and microbiota modulation	Moderate	DSS mouse study at 3 doses showed clinical and histologic improvement, decreased TNF-α/IL-1β/IL-6, enhanced Beclin-1/Atg5/LC3II/I, reduced p62 and PI3K/AKT/mTOR phosphorylation, with favorable shifts in microbiota	[28]
Butyrate-driven M2 macrophage polarization promotes goblet cells and mucus barrier repair	Moderate	In vitro and in vivo work showed butyrate induces M2 macrophages, increasing MUC2/SPDEF expression; WNT or ERK blockade blunted these effects; adoptive transfer of butyrate-primed M2 cells restored goblet cells and mucus after DSS	[29]
Butyrate-rich polymer nano-delivery (PSBA@Mag) enhances mucosal healing and drug efficacy in IBD models	Moderate	In DSS colitis and TNBS models, PSBA@Mag restored barrier proteins (ZO-1, occludin), reduced ROS and pro-inflammatory cytokines, promoted epithelial migration/healing, and normalized microbiota composition	[33]
Butyrate alleviates experimental colitis by inhibiting ferroptosis and modulating ERK/STAT3 signaling and the gut microbiota	Moderate	DSS mouse study showed reduced disease severity, decreased Fe^2+^ and MPO, increased GSH and SOD, enhanced ERK/STAT3 phosphorylation, and favorable modulation of gut microbiota following sodium butyrate treatment.	[37]
Overall, oral butyrate is a promising add-on for IBD, while enemas show mixed efficacy	Strong	Clinical and preclinical work concludes most oral-supplement studies show reduced inflammation and better remission maintenance, whereas enema results are variable	[17,18,20,23,25]

UC, ulcerative colitis; IBD, inflammatory bowel disease; MSB, microencapsulated sodium butyrate; UCDAI, Ulcerative Colitis Disease Activity Index; ESR, erythrocyte sedimentation rate; NLR, neutrophil-to-lymphocyte ratio; QoL, quality of life; hs-CRP, high-sensitivity C-reactive protein; DSS, dextran sulfate sodium; TNBS, 2,4,6-trinitrobenzenesulfonic acid; PI3K, phosphoinositide 3-kinase; AKT, protein kinase B; mTOR, mechanistic target of rapamycin; LC3, microtubule-associated protein 1 light chain 3; ROS, reactive oxygen species; MPO, myeloperoxidase; GSH, glutathione; SOD, superoxide dismutase; ERK, extracellular signal-regulated kinase; STAT3, signal transducer and activator of transcription 3; TNF-α, tumor necrosis factor-alpha; IL-1β, interleukin-1 beta; IL-6, interleukin-6; MUC2, mucin 2; SPDEF, SAM pointed domain-containing ETS transcription factor; WNT, Wingless/Integrated signaling pathway; ZO-1, zonula occludens-1; nNOS, neuronal nitric oxide synthase; ChAT, choline acetyltransferase; Fe^2+^, ferrous iron; SCFA, short-chain fatty acid.

## Data Availability

No new data were created or analyzed in this study. Data sharing is not applicable to this article.

## Supplementary Materials

The following supporting information can be downloaded at: https://www.mdpi.com/article/10.3390/medsci14040469/s1, Supplementary S1: PRISMA 2020 Checklist.
